# Treatment of periprosthetic joint infection – outcomes following algorithm-guided treatment at a multidisciplinary referral centre

**DOI:** 10.5194/jbji-11-113-2026

**Published:** 2026-02-12

**Authors:** Christian Merz, Jan Klaas, Rik Osinga, Parham Sendi, Richard Alexander Kuehl, Mario Morgenstern, Martin Clauss

**Affiliations:** 1 Center for Musculoskeletal Infections, University Hospital Basel, Basel, 4031, Switzerland; 2 Department of Orthopaedics, Balgrist University Hospital, Zurich, 8008, Switzerland; 3 Department of Orthopaedics and Trauma Surgery, University Hospital Basel, Basel, 4031, Switzerland; 4 Department of Plastic, Reconstructive, Aesthetic, and Hand Surgery, University Hospital Basel, Basel, 4031, Switzerland; 5 Institute for Infectious Diseases, University of Bern, Bern, 3001, Switzerland; 6 Department of Infectious Diseases, University Hospital Basel, Basel, 403, Switzerland

## Abstract

**Introduction**: This study evaluated early treatment outcomes and the management of complications in patients with periprosthetic joint infection (PJI) of the hip and knee treated at a specialized centre using an algorithm-guided multidisciplinary team (MDT) approach. **Methods**: This prospective cohort includes all consecutive patients treated for acute or chronic PJI between December 2019 and December 2022, with a minimum 1-year follow-up. PJI was defined according to the criteria of the European Bone and Joint Infection Society (EBJIS). Surgical decisions were based on a published treatment algorithm. The primary outcome was treatment success at 1 year after the last PJI surgery. **Results**: 106 patients were included according to prespecified criteria, with a median age of 74 years (IQR 66–82) and follow-up of 24 months (IQR 15–28). 79 patients (75 %) were referred from other institutions. 44 patients (42 %) were treated with debridement, antibiotics, and implant retention (DAIR); 17 (16 %) were treated with one-stage revision; and 45 (42 %) were treated with two-stage revision. 9 patients (8 %) needed plastic surgery for soft tissue reconstruction. The 1-year infection-free survival (Delphi-based consensus criteria) was 69 % (95 % CI: 60 %–78 %). Within 1 year, 14 (13.2 %) septic and 12 (11.3 %) aseptic failures occurred after a median of 0.5 months (IQR 0.4–1) and were successfully treated in most patients. 12 patients (11 %) died after a median of 0.8 months (IQR 0.3–2.8). **Conclusion**: Our results demonstrate the value of an algorithm-driven MDT approach as an effective strategy for managing complex PJI patients and PJI-surgery-related complications in a specialized referral centre for bone and joint infections.

## 1 Introduction

The ageing population in industrialized countries continues to drive an increasing demand for primary and revision total joint arthroplasties (Jones et al., 2025). This leads to a corresponding increase in periprosthetic joint infections (PJIs), a leading cause of morbidity and mortality after total joint arthroplasty (Kurtz et al., 2018; Ramos et al., 2025). Currently, the prevalence of PJI following primary total hip arthroplasty (THA) and total knee arthroplasty (TKA) ranges from 0.5 % to 2.4 %, with an increase of up to 20 % in patients with septic revision arthroplasties (Egerci et al., 2024)

Appropriate PJI management begins with an accurate diagnosis based on established definitions, followed by a patient-tailored surgical intervention and organism-specific antibiotic therapy (McNally et al., 2021; Osmon et al., 2013). Depending on the duration of symptoms, implant stability, soft tissue condition, and the causative bacteria, various treatment options for PJI are available.

While debridement, antibiotics, and implant retention (DAIR) is the surgical option for acute PJI (symptoms lasting less than 4 weeks), one- or two-stage prosthetic exchanges are typically recommended for chronic infection (symptoms lasting more than 4 weeks). The rationale for choosing between DAIR and exchange surgery was first defined over 20 years ago by Zimmerli et al. (2004) and has since become a standard for PJI treatment. Despite being well accepted, prospective controlled trials comparing the published algorithms to other approaches are still missing (Zimmerli and Trebse, 2023).

Various publications on how to define a successful outcome after treatment for PJI have been released, yet no consensus has been reached (Diaz-Ledezma et al., 2013; Fillingham et al., 2019). Therefore, the comparability of reported success rates after PJI treatment is limited, as they range widely from 55 % to 100 % (Corona et al., 2020; Born et al., 2016; Knoll et al., 2023; Zielinski et al., 2024). Evidence indicates that patients clearly benefit from algorithm-guided treatment within a multidisciplinary setting (Karczewski et al., 2019; Vuorinen et al., 2021).

The aim of this study was to assess early treatment outcomes and complication management after algorithm-guided PJI treatment in a complex referral cohort, with a detailed evaluation of septic and aseptic failures and consideration of varying definitions of treatment success.

## 2 Methods

### 2.1 Study design, setting

**Figure 1:**
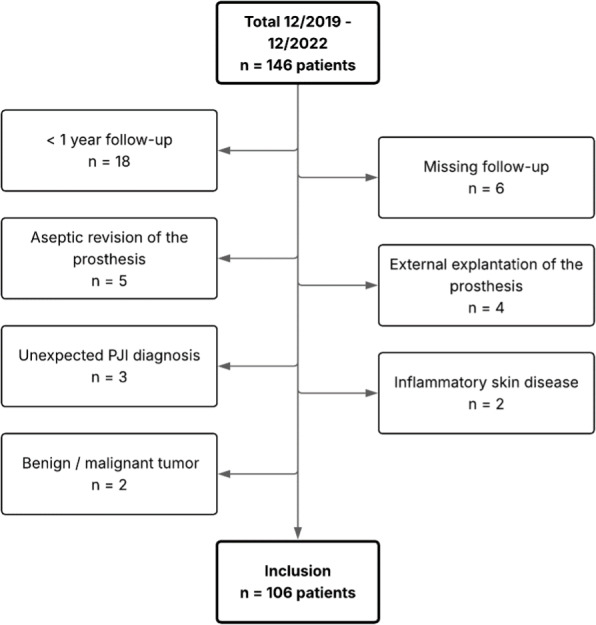
Patient exclusion flowchart.

In 2019, the Center for Musculoskeletal Infections (ZMSI) was established at the University Hospital of Basel to standardize the treatment of infections in orthopaedics and traumatology and to implement an MDT. For this retrospective analysis of a prospectively collected cohort, patients treated for acute or chronic PJI following primary or revision THA or TKA, in whom all prosthetic components were fully exchangeable, between December 2019 and December 2022 were included. PJI was defined according to the European Bone and Joint Infection Society (EBJIS) criteria (McNally et al., 2021). Patients with confirmed PJI treated exclusively at our institution and followed for at least 12 months after the last PJI-related surgery were included. Patients with unexpected positive intraoperative cultures or histology during presumed aseptic revision were excluded (Fig. 1).

The treatment plan for all PJI patients was defined by an interdisciplinary team comprising two experienced orthopaedic surgeons (MM, MC), one plastic–reconstructive surgeon (RO), and two infectious disease specialists (PS, RK). Surgical strategies included DAIR and one-stage and two-stage (with a short or long interval) procedures based on the principles defined by Zimmerli et al. (2004). All surgeries were performed or supervised by the two senior surgeons (MM, MC).

### 2.2 Surgical and antibiotic management

Surgical and antibiotic management followed the treatment algorithm by Zimmerli (2015). Briefly, DAIR was performed if clinical symptoms had lasted <3 weeks, the implant was stable, soft tissues were intact, and the pathogen was susceptible to agents active against biofilm. If these criteria were not met, a one-stage exchange was performed when soft tissues were intact or minimally compromised, and a two-stage exchange was performed otherwise. For two-stage procedures, a short interval of 2–4 weeks was used when no difficult-to-treat pathogen was present; a long interval of ≥8 weeks was applied when difficult-to-treat pathogens were identified.

When the pathogen was unknown at revision, empiric antibiotics consisted of amoxicillin–clavulanate or were tailored to the patient's risk profile and adapted once the pathogen was identified. Total antibiotic duration was 12 weeks after DAIR, one-stage exchange, and two-stage exchange with a short interval. For two-stage exchange with a long interval, antibiotics were administered for 6 weeks, followed by a 2-week antibiotic-free period before re-implantation. After re-implantation, antibiotics targeting the previous pathogen were restarted until final tissue culture results were available. If cultures remained negative, antibiotics were discontinued; if the same or a new pathogen was isolated, a full 12-week course was initiated.

Preoperative antibiotic prophylaxis was given according to in-house guidelines and was not withheld before tissue sampling. In staphylococcal PJI, rifampicin was added whenever a definitive implant was in place (after DAIR, after one-stage exchange, and after re-implantation in two-stage exchange with a short interval). Rifampicin was not used during the interval while a spacer was in situ. Local antibiotics (gentamicin, vancomycin, or clindamycin) were incorporated into spacers during two-stage exchange intervals, but not in other procedures. Spacers in the knee were designed as mobile spacers using commercial spacer models (Heraeus, Wehrheim, Germany), in cases with insufficient collateral ligaments or after the explantation of hinged static knee spacers. Regarding the hip, cement spacers were used whenever possible. Other local antibiotic carriers were not used routinely.

Re-implantation was carried out using off-the-shelf revision systems, with implant choice determined by the operating surgeon according to stability requirements. As the algorithm does not rely on adding local antibiotics during replantation, the vast majority of revision THA was done using uncemented fluted revision stems (Wagner SL, Zimmer, Winterthur, CH) combined with various cup systems. In revision TKA, the Legion revision system and the RT revision system (both Smith + Nephew, Winterthur, CH) were mainly used.

### 2.3 Follow-up

Follow-up examinations were prospectively scheduled at 6 weeks, 3 months, 6 months, 1 year, and 2 years after the last PJI surgery.

### 2.4 Outcome

Endpoints for survival analysis were defined as septic failures (secondary infection or chronic PJI), aseptic failures (wound or mechanical complications in which revision surgery revealed no macroscopic signs of infection and no pathogen was identified by microbiological or pathological analysis), PJI-related mortality, new PJI, and suppressive therapy (planned or unplanned) without curative intention.

The primary outcome was treatment success 1 year after the last PJI surgery. We employed two different outcome definitions and compared them.

Outcome A was defined according to the published Delphi-based consensus criteria (Diaz-Ledezma et al., 2013) as follows:

1. Infection eradication, characterized by a healed wound without fistula, drainage, or pain, and no infection recurrence caused by the same organism.
2. No occurrence of PJI-related mortality.
3. Antibiotic therapy stopped for at least 6 months.
4. No subsequent surgical intervention after for infection after re-implantation surgery.

Outcome B was defined as an extended, patient-centred treatment success. It differs from Outcome A only in criterion 4, which allows:

1. Early postoperative DAIR or wound washout (within 4 weeks) for prolonged wound leakage, with the same pathogen, a new pathogen, or negative cultures.
2. Later ( >4 weeks) aseptic or mechanical complications requiring revision or arthroplasty exchange.

### 2.5 Statistical analysis

Survival analyses were conducted using Kaplan–Meier estimates for outcome definitions (A and B). Patients treated with a planned non-curative approach requiring suppressive therapy after PJI surgery, those who developed new hematogenous PJIs (defined as infection caused by a different pathogen after a symptom-free interval) within 1 year, and patients who died unrelated to PJI were not considered treatment failures and were censored for survival analysis.

To compare early failure rates between hip and knee PJIs, a weighted log-rank test (Tarone–Ware test) was employed. Differences in survival by treatment type were evaluated using Cox proportional hazards models. All analyses were performed using R version 4.4.2. A p value of <0.05 was considered statistically significant.

## 3 Results

### 3.1 Study population

We screened 146 patients for eligibility in this study, of which 40 were excluded due to prespecified criteria. Hence, 106 patients, one joint per patient, were included with a median age of 74 (interquartile range (IQR) 66–82) years (Table 1). The median follow-up was 24 (IQR 15–28) months.

**Table 1:** Patient characteristics

		n	%
Age (years)	<55	7	6.6
	55–64	17	16
	65–75	32	30.2
	>75	50	47.2
Sex	Female	48	45.3
	Male	58	54.7
ASA classification	I	5	4.7
	II	14	13.2
	III	79	74.5
	IV	8	7.5
Charlson score	0–2	59	55.7
	3–4	30	28.3
	5–6	14	13.2
	>6	3	2.8
Index arthroplasty	Study centre	27	25.5
	External centre	79	74.5
Cause of infection	Exogenous	42	39.6
	Haematogenous	41	38.7
	Unknown	23	21.7
Affected joint	Hip	57	53.8
	Knee	49	46.2
Type of arthroplasty	Primary	59	55.7
	Revision	47	44.3

44 (41.5 %) patients were treated with DAIR, 17 (16 %) were treated with a one-stage approach, and 45 (42.5 %) were treated with a two-stage approach. Overall, 9 patients (8.5 %) needed soft tissue reconstruction at PJI surgery (one-stage n=2 , two-stage n=7 ), all of which were TKAs with prior external revisions. Details on the treatment strategy in relation to the implant type and affected joint are presented in Table S1 in the Supplement.

Among the revision arthroplasties, the median number of previous revisions was 2 (IQR 1–3, total n=102 revisions). Revisions were performed externally in 40 patients (85.1 %).

At the time of admission, 37 (34.9 %) patients were under ongoing antibiotic therapy. Of these, 29 (27.4 %) had a PJI confirmed by joint aspiration, and 8 (7.5 %) presented with a septic clinical condition. 15 (14.2 %) patients were referred following unsuccessful revision procedures for PJI performed at external institutions.

### 3.2 Pathogen distribution

15 patients (14.2 %) had polymicrobial PJI involving up to four different pathogens, 6 (5.7 %) presented with culture-negative PJI, and 2 (5.3 %) were diagnosed with fungal PJI. For details on pathogen distribution, see Figs. S1 and S2 in the Supplement.

### 3.3 Treatment outcome

#### 3.3.1 Survival analysis

**Figure 2:**
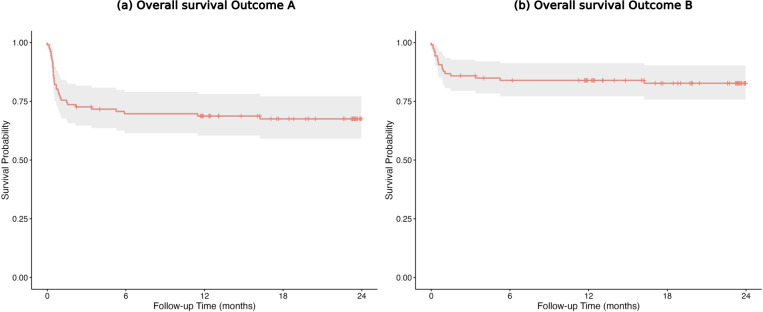
**(a, b)** Infection-free 1-year survival overall for Outcome A and Outcome B.

Infection-free 1-year survival was 68.7 % (95 % confidence interval (CI): 60–78) for Outcome A and 83.9 % (95 % CI: 77–91) for Outcome B (Fig. 2a, b).

**Figure 3:**
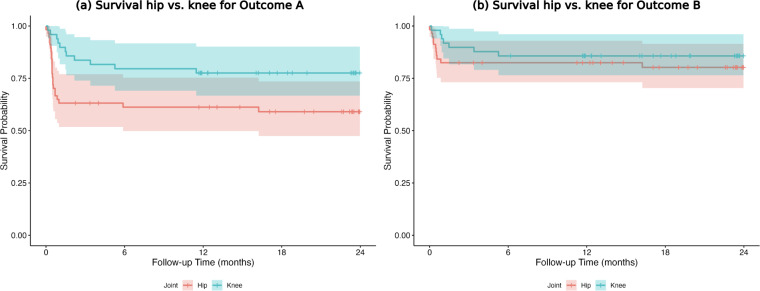
**(a, b)** Infection-free 1-year survival hip vs. knee for Outcome A and Outcome B.

For Outcome A, 1-year survival was higher for knees than for hips (77.6 % versus 61.2 %; HR, 0.47; p=0.041 ); no difference was observed for Outcome B (85.7 % versus 82.5 %) (Fig. 3a, b).

**Figure 4:**
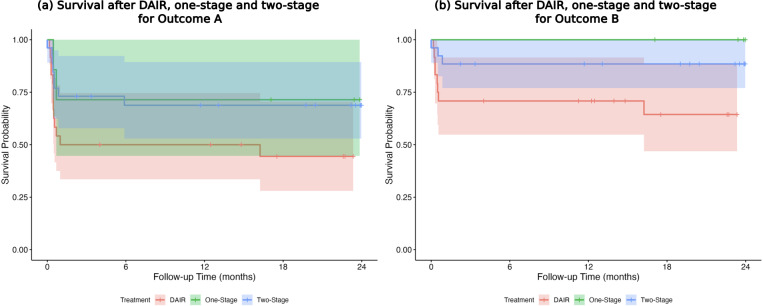
**(a, b)** Infection-free 1-year survival after DAIR, one-stage revision, and two-stage revision for Outcome A and Outcome B.

Infection-free 1-year survival for DAIR, one-stage revision, and two-stage revision was 56.7 % (95 % CI: 44–74), 76.5 % (95 % CI: 59–100), and 77.6 % (95 % CI: 66–91) for Outcome A and 70.4 % (95 % CI: 58–85), 100 % (95 % CI: 100–100), and 91.1 % (95 % CI: 83–100) for Outcome B respectively (Fig. 4a, b).

For Outcome A, there was a trend towards better outcomes after one-stage (HR = 0.46, p=0.156 ) and two-stage revision (HR = 0.47, p=0.056 ) compared to DAIR. For Outcome B, two-stage treatment offered significantly better survival compared to DAIR (HR = 0.28, p=0.0258 ). Due to missing events, significance was not estimable for one-stage treatment.

For infection-free 1-year survival by joint after DAIR, one-stage revision, and two-stage revision for Outcome A and Outcome B, see Text S1 in the Supplement.

At the end of the observation period, regardless of the number of septic or aseptic revisions following PJI surgery, 89.4 % (76 of 85) of the surviving patients were cured of infection. Omitting the 7 patients with planned suppressive therapy, 94.9 % (75 out of 79) of the surviving patients were free of infection (Figs. S3–S5).

#### 3.3.2 Septic failure

Within the first year, we observed 14 (13.2 %) septic complications (secondary infection, chronic PJI) after a median of 0.5 months (IQR 0.4–1 months) after PJI surgery (Table 2).

**Table 2:** Septic failures within 1 year.

	Initial treatment	n	Days after surgery	Cause of failure	Treatment of failure	Outcome of failure treatment at last follow-up
Hip	DAIR	7	30	Secondary infection	DAIR	Infection controlled
			9	Secondary infection	DAIR	Infection controlled
			13	Secondary infection	Two-stage	Death <1 year
			7	Chronic PJI	DAIR	Infection controlled
			14	Chronic PJI	DAIR	Infection controlled
			9	Chronic PJI	Two-stage	New PJI
			17	Chronic PJI	Two-stage	Infection controlled
	Two-stage (short)	1	12	Secondary infection	DAIR	Infection controlled
	Two-stage (long)	2	11	Secondary infection	DAIR	Infection controlled
			16	Chronic PJI	DAIR	Infection controlled
Knee	DAIR	4	16	Chronic PJI	DAIR	Suppressive therapy (tumour prosthesis)
			160	Chronic PJI	One-stage	Infection controlled
			32	Chronic PJI	One-stage	Infection controlled
			45	Chronic PJI	Two-stage	Infection controlled

Overall, 11 of the 14 (78.6 %) re-revision patients were successfully cured.

#### 3.3.3 Aseptic failure

Within the first year, we observed 12 (11.3 %) aseptic complications after a median of 0.5 (IQR 0.4–1) months. 9 (8.5 %) had wound complications requiring surgical revision after a mean of 0.8 months (standard deviation (SD) ± 0.6 months) following PJI revision (Table 3).

**Table 3:** Aseptic failures (wound complications) within 1 year.

	Initial treatment	n	Days after surgery	Treatment of failure (reason)	Outcome of failure treatment at last follow-up
Hip	DAIR	4	21	Wound revision (leakage)	Infection controlled
			14	DAIR (leakage)	Infection controlled
			15	DAIR (leakage)	Death <1 year
			14	DAIR (leakage)	Infection controlled
	One-stage	2	14	Wound revision (leakage)	Infection controlled
			21/492	2× plastic revision (seroma cavity with secondary fistula)	Infection controlled
Knee	DAIR	1	47	Wound revision (necrosis)	Infection controlled
	One-stage	1	66	Plastic revision (failure extensor mechanism)	Death <1 year
	Two-stage (short)	1	10/45	2× plastic revision (failure extensor mechanism)	Infection controlled

Furthermore, we observed three (2.8 %) mechanical complications requiring surgical revision after a mean of 6.2 months (SD ± 4.5 months): one hip dislocation following two-stage revision necessitating cup revision, one aseptic loosening of a hip stem after two-stage revision, and one aseptic loosening of both components following one-stage revision of a TKA. Additionally, one hip dislocation occurring 2 months after two-stage revision was managed conservatively.

Overall, 10 of the 12 (83.3 %) aseptic re-revision patients were successfully cured of PJI.

#### 3.3.4 PJI-related mortality

Within the first year, 12 (11.3 %) patients died after a mean of 0.8 months (IQR 0.3–2.8 months), including 5 who died after explantation during a two-stage approach. Death due to infection was likely in 8 patients and occurred within 1 month of PJI surgery (Table 4).

**Table 4:** Death within 1 year.

		n	Days after	Cause	PJI-surgery
			surgery		related
Hip	DAIR	3	5	Cardiac arrest	Likely
			13	Pulmonary complications	Likely
			123	Contralateral femoral neck fracture	Unlikely
	Two-stage (short)	3	16	Respiratory insufficiency	Likely
			0	Severe sepsis	Likely
			68	Septic abdominal complications	Unlikely
	Two-stage (long)	2	26	Unknown during rehab	Likely
			102	Unknown	Unlikely
Knee	DAIR	2	25	Severe sepsis	Likely
			28	Severe urosepsis	Likely
	One-stage	1	188	Severe sepsis after colonic ischemia	Unlikely
	Two-stage (short)	1	4	Encephalopathy	Likely

#### 3.3.5 Planned suppressive therapy and new PJI within 1 year

7 out of 106 patients (6.6 %) were treated with planned suppressive therapy after a DAIR procedure without an initial intention for a curative approach (Table S2).

Within the first year, we observed six (5.7 %) new hematogenous PJIs with a new pathogen after a median of 4.6 months (IQR 2.2–6.1 months) after an, until then, uneventful follow-up . In three cases, bacteremia was associated with diabetic ulcerations; one case involved bacteremia related to complications of abdominal surgery. In two cases, the same pathogen was identified with a different morphotype. Five were treated with a DAIR, and one was treated with a two-stage approach due to poor soft tissue (Table S3).

## 4 Discussion

In the setting of a tertiary referral centre, we found an infection-free 1-year survival ranging from 69 % (Delphi criteria) up to 84 % depending on the definition used. In most patients within our complex cohort, a single application of the treatment algorithm was sufficient to control severe infections and their associated complications. Both septic and aseptic complications were successfully managed, with 89 % of surviving patients remaining infection-free at the final follow-up.

A septic complication rate of 13.2 % is acceptable, particularly considering that in our cohort many patients were severely ill or had undergone prior external revisions (Resl et al., 2024; Lee et al., 2023). Most septic complications occurred within the first 4 postoperative weeks, and we were able to cure infection in 78.6 % of these patients. There is no consensus on how to classify new infections after PJI surgery. Therefore, these six cases (5.7 %) were censored in our survival analysis; in two cases, detection of the same pathogen with a different morphotype suggests a likelihood of recurrence (Heckmann et al., 2025; Piuzzi et al., 2025).

Our proactive strategy to surgically manage persistent wound drainage ( > 10–14 d) was associated with an aseptic revision rate of 8.5 %. Even though these interventions are considered failures according to Delphi criteria, a 77.8 % healing rate was achieved, indicating a clear clinical benefit.

Aseptic loosening and hip dislocations were rare complications in our cohort. There are conflicting data in the literature concerning the prevalence of aseptic loosening following successful treatment of PJI (Kienzle et al., 2020; Clauss et al., 2020). As there seems to be no increased risk of aseptic failure after DAIR, it remains at least debatable whether aseptic loosening results from PJI treatment itself or represents an inherent risk associated with revision arthroplasty, often complicated by severe bone loss.

In our cohort, 11 % of the patients died within the first year, with 75 % of those dying within 4 weeks after PJI surgery, suggesting a likely association. Especially when accompanied by sepsis, PJI is associated with substantial morbidity and mortality (Baertl et al., 2024; Mundi et al., 2024; Puelacher et al., 2025), with recent data indicating that 3-year post-PJI mortality approaches the 5-year survival rates of common cancers (Ramos et al., 2025).

Comparison of the THA and TKA cohorts revealed a significantly better 1-year survival following PJI in the TKA group, based on the Delphi criteria (Outcome A), but not for Outcome B. To us, this result is of limited significance and highlights the problem of inconsistent definitions of treatment success.

The highest failure rate was found for the DAIR cohort. In line with our findings and the existing literature, elevated failure rates following DAIR are common, underscoring the persistent clinical challenge of appropriate patient selection (Huffaker et al., 2022; Liukkonen et al., 2024).

Chronic PJIs with a pathogen susceptible to biofilm-active antibiotics and presenting with only minimal soft tissue compromise were treated with a one-stage exchange. This selection resulted in excellent treatment outcomes, comparable to other series employing the same algorithm (Born et al., 2016; Ilchmann et al., 2016). It remains unclear whether results remain as good when selection is less strict, but there seems to be a shift from two-stage to one-stage revision surgery in the literature (Matar et al., 2021; Razii et al., 2021).

The two-stage subgroup demonstrated low rates of septic and aseptic complications; however, the requirement for at least two procedures increases the risk of morbidity and mortality and contributes to higher healthcare costs (Alt et al., 2025; Barton et al., 2020). Despite this, the two-stage approach remains the gold standard, e.g. for difficult-to-treat pathogens or patients with critical soft tissue conditions.

This study is limited by its retrospective design, lack of randomization, small surgical subgroup sizes, and short follow-up, which preclude conclusions regarding potential failure mechanisms. Additional limitations include referral-centre selection bias, strict one-stage selection, short follow-up for late hematogenous events, and dependence of success rates on outcome definitions.

## 5 Conclusion

The lack of a unified definition of treatment success after PJI surgery remains a major issue. Unified criteria to define treatment success, as currently in development, are crucial to making data more comparable in the future. Centralized, multidisciplinary care and the consistent application of established treatment principles by experienced teams are likely to be beneficial for improving outcomes in these complex patients.

## Supplement

10.5194/jbji-11-113-2026-supplement The supplement related to this article is available online at https://doi.org/10.5194/jbji-11-113-2026-supplement.

## Data Availability

All raw data can be provided by the corresponding authors upon request.
