# Supplementary material for: Treatment of periprosthetic joint infection – outcomes following algorithm-guided treatment at a multidisciplinary referral centre
Source: J Bone Jt Infect. 2026 Feb 12;11(1):113–21. doi: 10.5194/jbji-11-113-2026 (PMC12919659; doi:10.5194/jbji-11-113-2026)
Supplement: The supplement related to this article is available online at https://doi.org/10.5194/jbji-11-113-2026-supplement. [file jbji-11-113-2026-supplement.zip › Table S1.pdf]

Table S1: Treatment strategy in relation to type of implant and affected joint

Treatment strategy			Type of implant			
			Primary		Revision	
	n	%	n	%	n	%
**Hip**	57	53.8	33	57.9	24	42.1
DAIR	24	42.1	17	51.5	7	29.2
One-stage	7	12.3	5	15.5	2	8.3
Two-stage short	6	10.5	4	12.1	2	8.3
Two-stage long	20	35.1	7	21.2	13	54.2
**Knee**	49	46.2	26	53.1	23	46.9
DAIR	20	40.8	18	69.2	2	8.7
One-stage	10	20.4	2	11.1	8	34.8
Two-stage short	7	14.3	2	11.1	5	21.7
Two-stage long	12	24.5	4	15.4	8	34.8
