# Supplementary material for: Treatment of periprosthetic joint infection – outcomes following algorithm-guided treatment at a multidisciplinary referral centre
Source: J Bone Jt Infect. 2026 Feb 12;11(1):113–21. doi: 10.5194/jbji-11-113-2026 (PMC12919659; doi:10.5194/jbji-11-113-2026)
Supplement: The supplement related to this article is available online at https://doi.org/10.5194/jbji-11-113-2026-supplement. [file jbji-11-113-2026-supplement.zip › Text S1.pdf]

Text S1: Survival by joint after DAIR vs. one-stage vs. two-stage for Outcome A and B

One-year survival for DAIR, one-stage, and two-stage revision in hip-PJI was 50% (95% CI: 34 – 75), 71.4% (95% CI: 45 - 100), and 68.8% (95% CI: 53 - 89) for infection eradication (outcome A) and 70.8% (95% CI: 55 - 92), 100% (95% CI: 100 - 100), and 88.5% (95% CI: 77 - 100) for outcome B respectively (Figure 5 (a, b)).

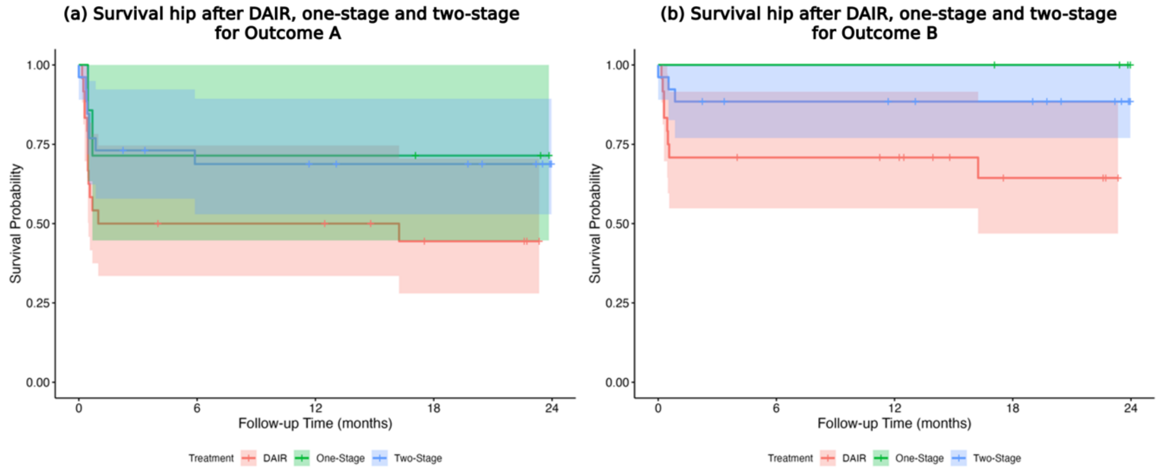

Figure 5 (a, b): Survival hip after DAIR vs. one-stage vs. two-stage Outcome A and B

One-year survival after treatment with DAIR, one-stage, and two-stage procedures in knee-PJI was 65% (95% CI: 47 - 90), 77.8% (95% CI: 56 - 100), and 94.1% (95% CI: 84 - 100) or 70.0% (95% CI: 53 - 93), 100% (95% CI: 100 - 100), and 100% (95% CI: 71 - 100) respectively (Figure 6 (a, b)).

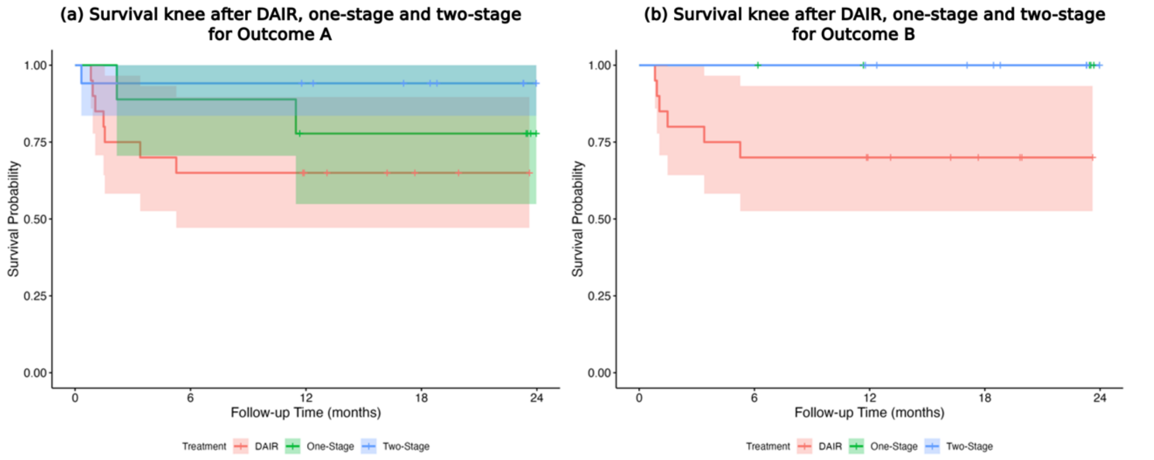

Figure 6 (a, b): Survival knee after DAIR vs. one-stage vs. two-stage Outcome A and B
