# Supplementary material for: Treatment of periprosthetic joint infection – outcomes following algorithm-guided treatment at a multidisciplinary referral centre
Source: J Bone Jt Infect. 2026 Feb 12;11(1):113–21. doi: 10.5194/jbji-11-113-2026 (PMC12919659; doi:10.5194/jbji-11-113-2026)
Supplement: The supplement related to this article is available online at https://doi.org/10.5194/jbji-11-113-2026-supplement. [file jbji-11-113-2026-supplement.zip › Table S3.pdf]

Table S3: New PJI within one year

		n	Days after surgery	Treatment	Outcome at last follow-up
Hip	Two-stage (long)	1	209	DAIR	Cured
Knee	DAIR	2	184	DAIR	Cured
			167	Two-stage	Cured
	Two-stage (long)	3	20	DAIR	Cured
			116	DAIR	Suppressive therapy (C. albicans and fistula)
			68	DAIR	Cured
