# Supplementary material for: Treatment of periprosthetic joint infection – outcomes following algorithm-guided treatment at a multidisciplinary referral centre
Source: J Bone Jt Infect. 2026 Feb 12;11(1):113–21. doi: 10.5194/jbji-11-113-2026 (PMC12919659; doi:10.5194/jbji-11-113-2026)
Supplement: The supplement related to this article is available online at https://doi.org/10.5194/jbji-11-113-2026-supplement. [file jbji-11-113-2026-supplement.zip › jbji-11-113-2026-supplement-title-page.pdf]

Supplement of J. Bone Joint Infect., 11, 113–121, 2026
https://doi.org/10.5194/jbji-11-113-2026-supplement
© Author(s) 2026. CC BY 4.0 License.

Supplement of

Treatment of periprosthetic joint infection – outcomes following algorithm-guided treatment at a multidisciplinary referral centre

Christian Merz et al.

Correspondence to: Martin Clauss (martin.clauss@usb.ch)

- jbji-11-113-2026-supplement-title-page.pdf
- Figure S1.pdf
- Figure S2.pdf
- Figure S3.pdf
- Figure S4.pdf
- Figure S5.pdf
- Table S1.pdf
- Table S2.pdf
- Table S3.pdf
- Text S1.pdf

The copyright of individual parts of the supplement might differ from the article licence.
