# Supplementary material for: Treatment of periprosthetic joint infection – outcomes following algorithm-guided treatment at a multidisciplinary referral centre
Source: J Bone Jt Infect. 2026 Feb 12;11(1):113–21. doi: 10.5194/jbji-11-113-2026 (PMC12919659; doi:10.5194/jbji-11-113-2026)
Supplement: The supplement related to this article is available online at https://doi.org/10.5194/jbji-11-113-2026-supplement. [file jbji-11-113-2026-supplement.zip › Table S2.pdf]

Table S2: Planned suppressive therapy

		n	Rationale for suppressive therapy	Outcome overall at last follow-up
Hip	DAIR	4	Microbial load reduction in sepsis (ASA IV)	Alive
			Microbial load reduction in sepsis (ASA IV)	Alive
			99-year-old patient with chronic fistula (ASA III)	Alive
			Chronic PJI of the right knee with fistula leading to PJI of the left hip. Successful definitive revision of the left hip following thigh amputation (ASA III)	Cured
Knee	DAIR	3	Microbial load reduction in sepsis (ASA IV)	Alive
			Palliative situation pleural mesothelioma (ASA III)	Death >1 year
			93-year-old patient with recurrent endocarditis (ASA III)	Death >1 year
