# Supplementary figures and images for: Treatment of periprosthetic joint infection – outcomes following algorithm-guided treatment at a multidisciplinary referral centre
Source: J Bone Jt Infect. 2026 Feb 12;11(1):113–21. doi: 10.5194/jbji-11-113-2026 (PMC12919659; doi:10.5194/jbji-11-113-2026)

Figure S5: Flowchart PJI knee

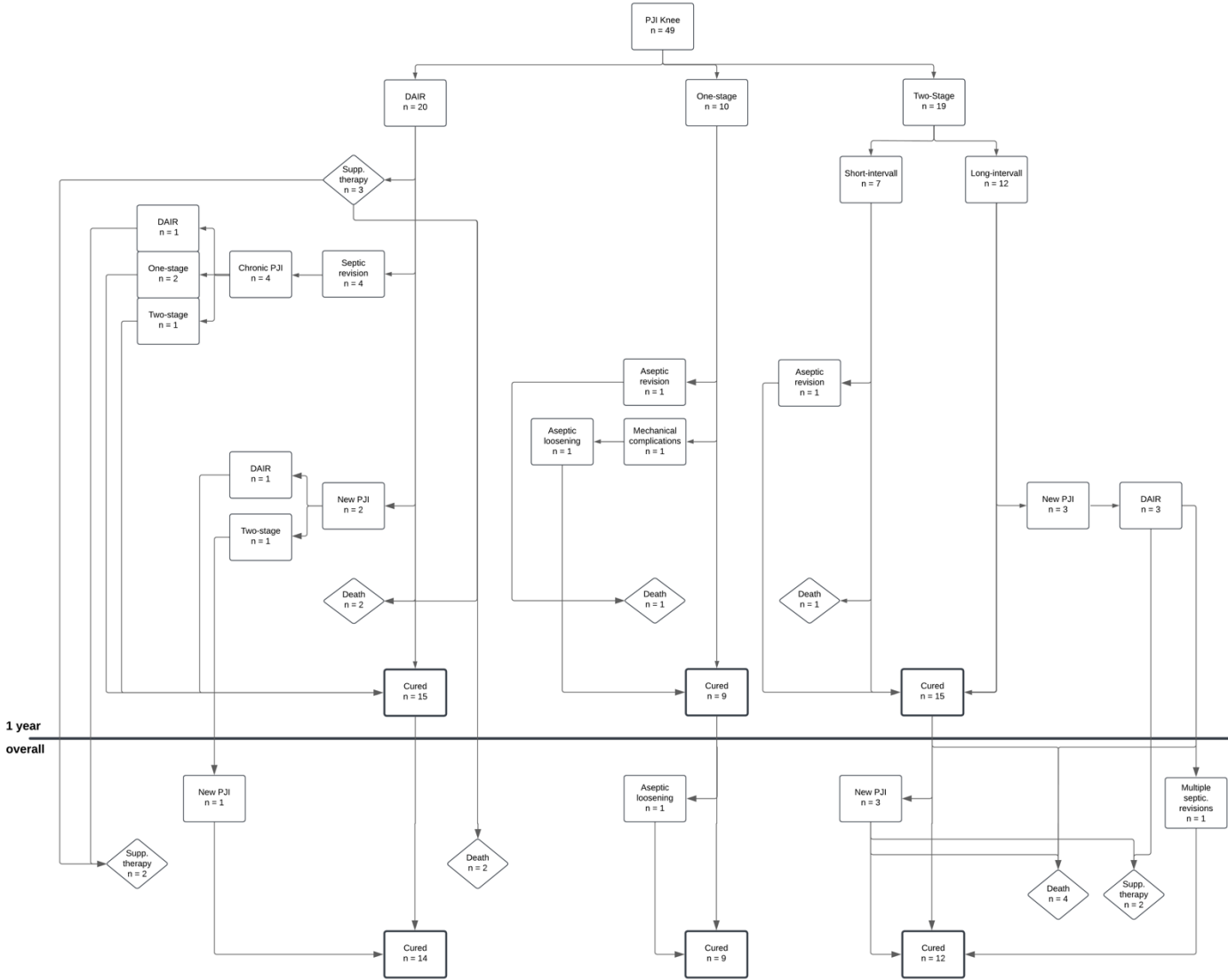

Supplement: The supplement related to this article is available online at https://doi.org/10.5194/jbji-11-113-2026-supplement. [file jbji-11-113-2026-supplement.zip › Figure S5.pdf]

**Figure S2: Pathogens knee**

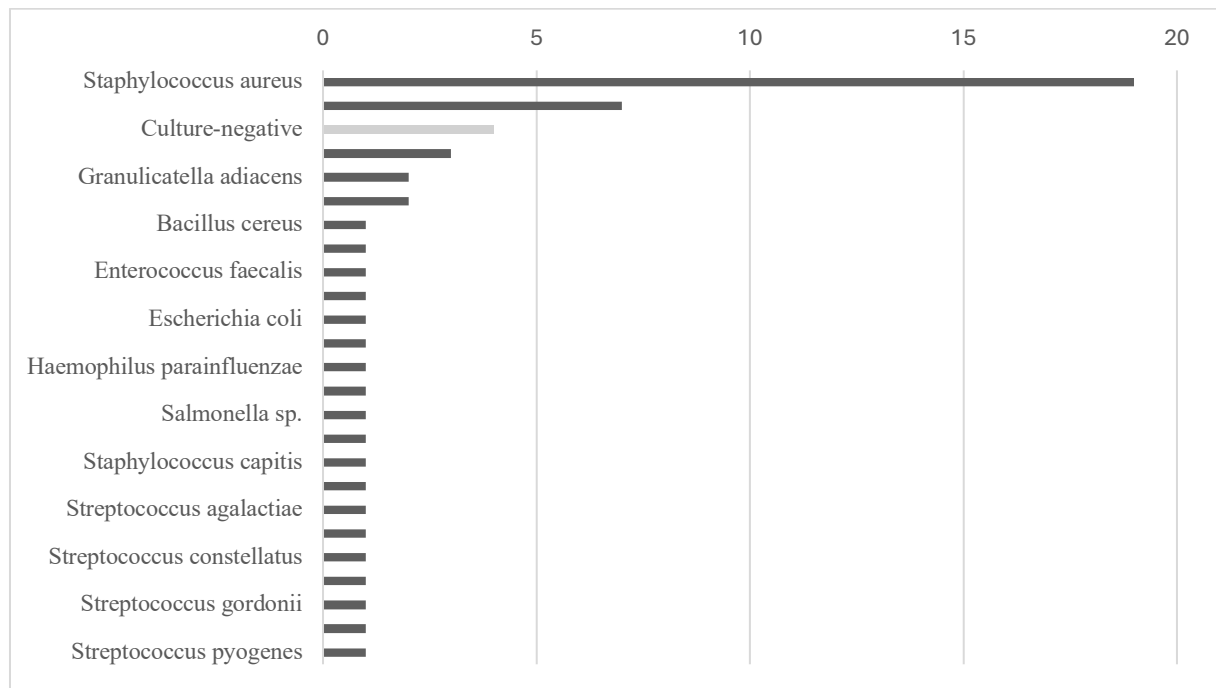

Supplement: The supplement related to this article is available online at https://doi.org/10.5194/jbji-11-113-2026-supplement. [file jbji-11-113-2026-supplement.zip › Figure S2.pdf]

**Figure S1: Pathogens hip**

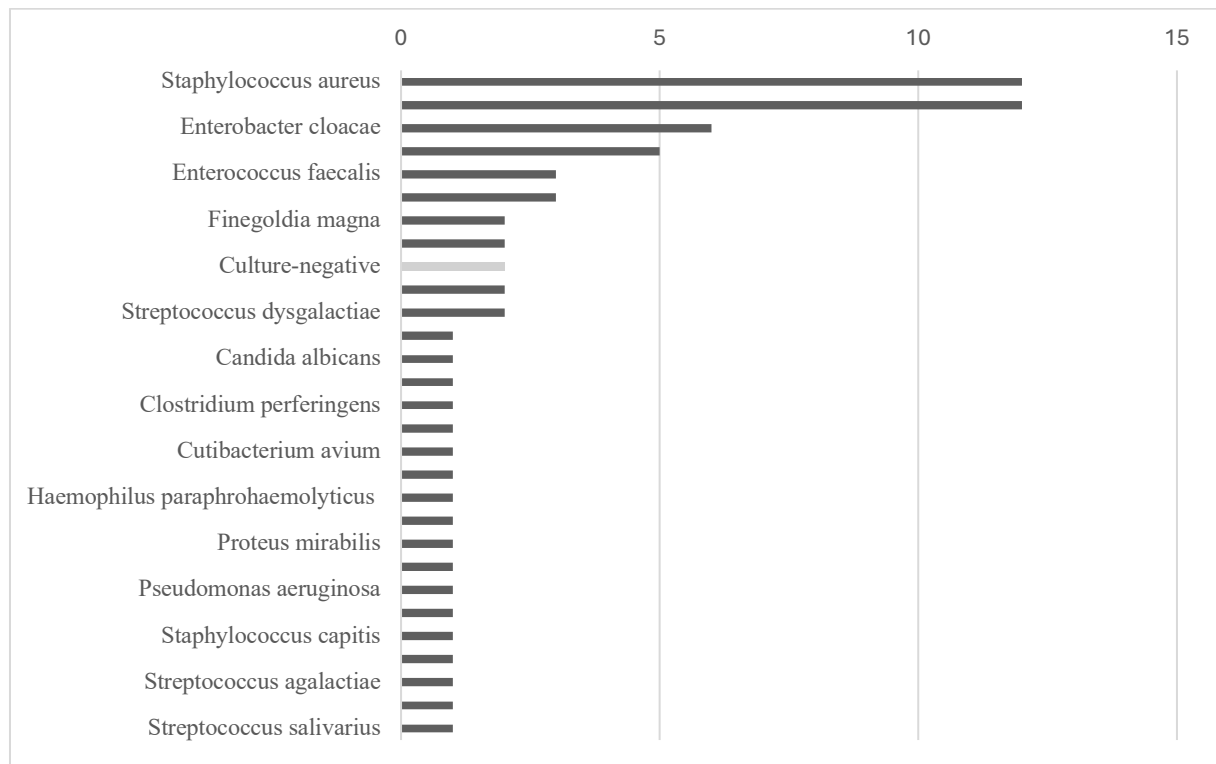

Supplement: The supplement related to this article is available online at https://doi.org/10.5194/jbji-11-113-2026-supplement. [file jbji-11-113-2026-supplement.zip › Figure S1.pdf]

Figure S4: Flowchart PJI hip

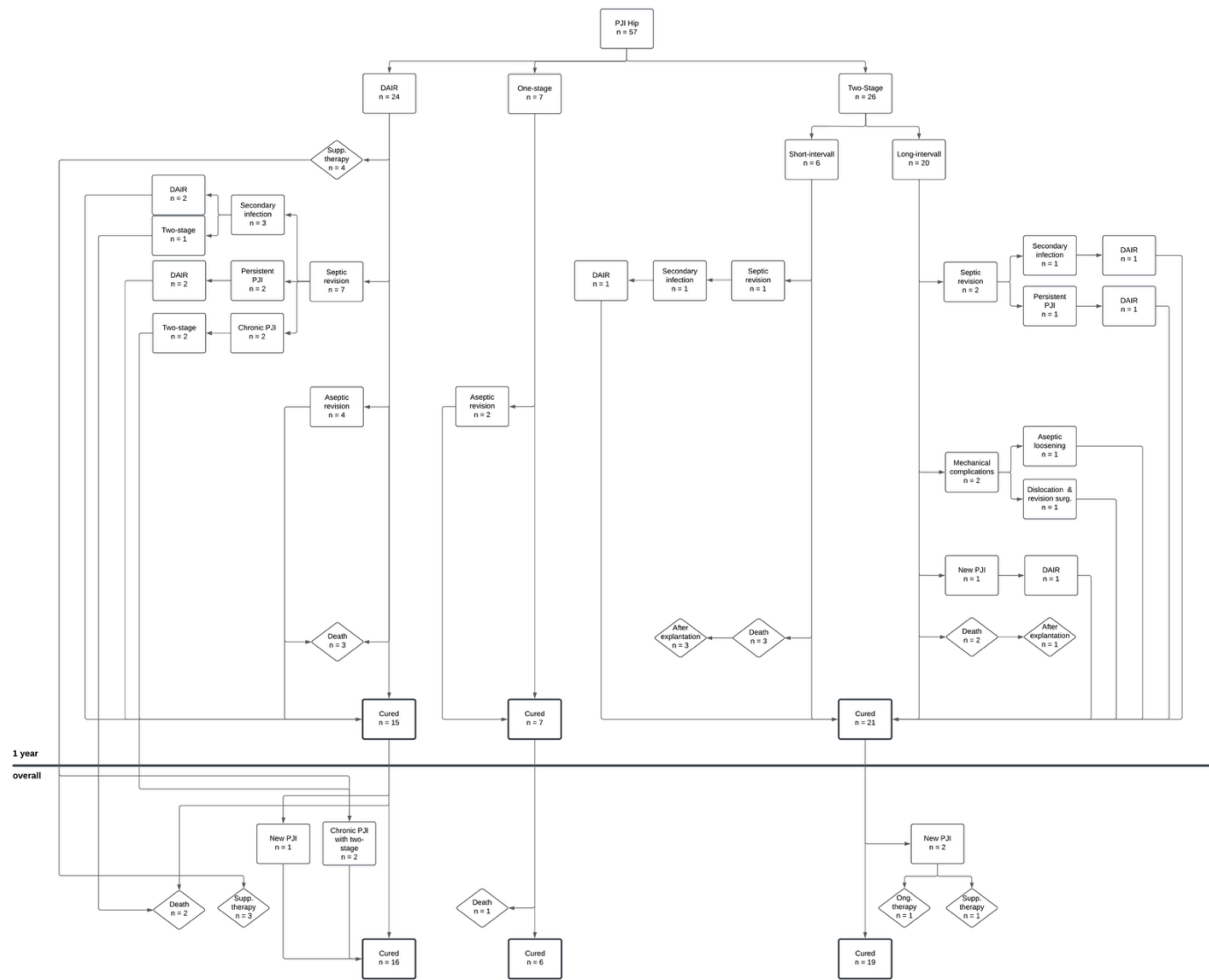

Supplement: The supplement related to this article is available online at https://doi.org/10.5194/jbji-11-113-2026-supplement. [file jbji-11-113-2026-supplement.zip › Figure S4.pdf]

**Figure S3: Flowchart PJI all**

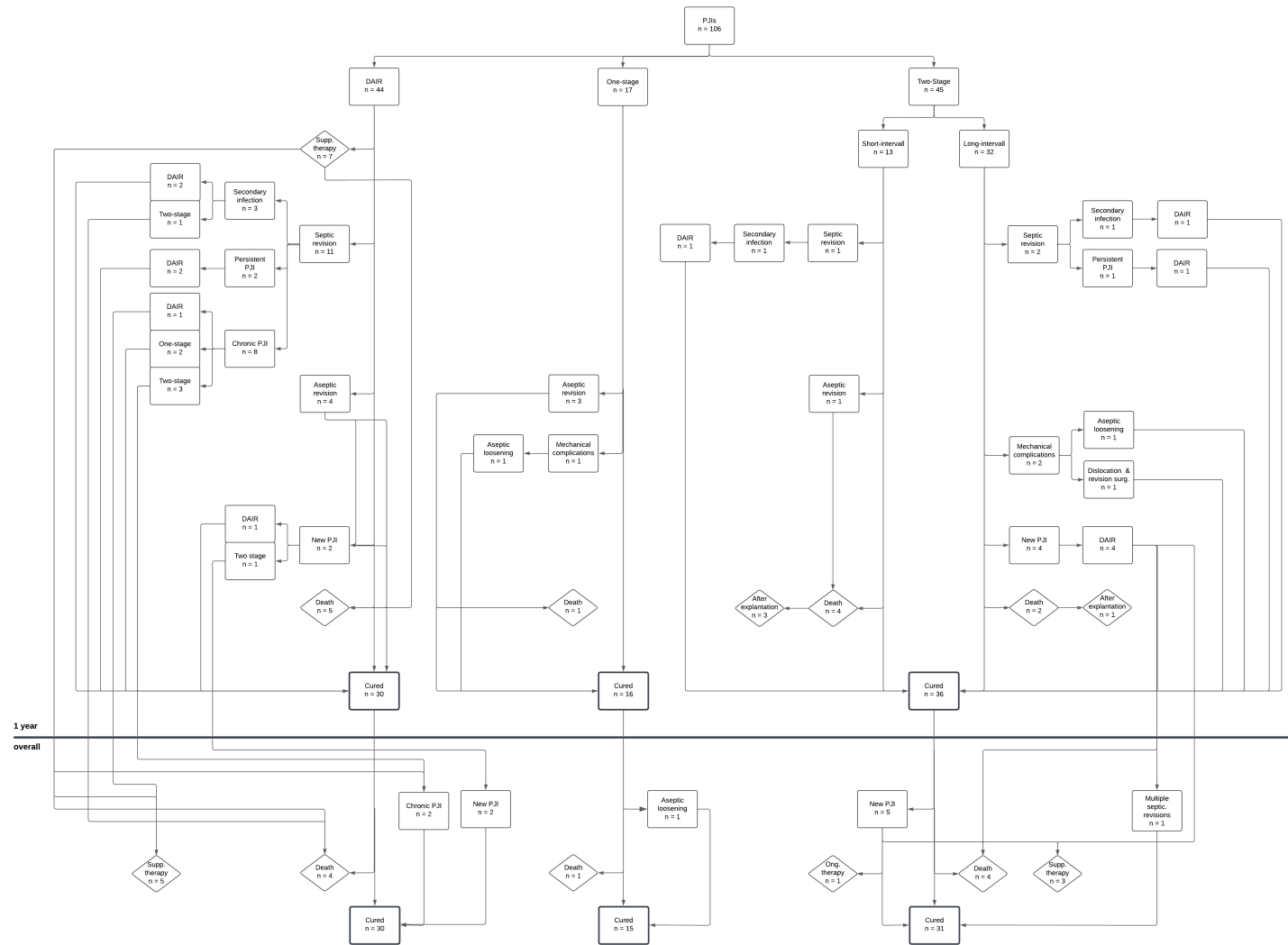

Supplement: The supplement related to this article is available online at https://doi.org/10.5194/jbji-11-113-2026-supplement. [file jbji-11-113-2026-supplement.zip › Figure S3.pdf]
